# Development of a New Molecular Marker for the Resistance to Tomato Yellow Leaf Curl Virus

**DOI:** 10.1155/2018/8120281

**Published:** 2018-07-17

**Authors:** Adedze Yawo Mawunyo Nevame, Lu Xia, Chofong Gilbert Nchongboh, Muhammad Mahmudul Hasan, Md. Amirul Alam, Li Yongbo, Zhang Wenting, He Yafei, Reza Mohammad Emon, Mohd Razi Ismail, Andrew Efisue, Sun Gang, Li Wenhu, Si Longting

**Affiliations:** ^1^Molecular Biology Laboratory of Jiangsu Green Port Modern Agriculture Development Company, Nancai Township Road No. 1, Suqian City, Jiangsu Province 223800, China; ^2^Catholic University Institute of Buea, P.O. Box 563, Buea, Cameroon; ^3^Bangladesh Institute of Nuclear Agriculture, BAU Campus, Mymensingh 2202, Bangladesh; ^4^Institute of Tropical Agriculture and Food Security, Universiti Putra Malaysia (UPM), 43400 Serdang, Selangor, Malaysia; ^5^Faculty of Sustainable Agriculture, Horticulture and Landscaping Program, Universiti Malaysia Sabah, Sandakan Campus, 90509 Sandakan, Sabah, Malaysia; ^6^College of Life Science, Nanjing Agricultural University, Nanjing 210095, China; ^7^Departments of Crop and Soil Science, University of Port Harcourt, Port Harcourt, Nigeria

## Abstract

*Tomato yellow leaf curl virus* (TYLCV) responsible for tomato yellow leaf curl disease (TYLCD) causes a substantial decrease in tomato (*Solanum lycopersicum* L.) yield worldwide. The use of resistant variety as a sustainable management strategy has been advocated. Tremendous progress has been made in genetically characterizing the resistance genes (*R* gene) in tomato. Breeding tomato for TYLCV resistance has been based mostly on* Ty-3 *as a race-specific resistance gene by introgression originating from wild tomato species relatives. Improvement or development of a cultivar is achievable through the use of marker-assisted selection (MAS). Therefore, precise and easy use of gene-targeted markers would be of significant importance for selection in breeding programs. The present study was undertaken to develop a new marker based on* Ty-3* gene sequence that can be used for MAS in TYLCV resistant tomato breeding program. The new developed marker was named ACY. The reliability and accuracy of ACY were evaluated against those of* Ty-3* linked marker P6-25 through screening of commercial resistant and susceptible tomato hybrids, and genetic segregation using F2 population derived from a commercial resistant hybrid AG208. With the use of bioinformatics and DNA sequencing analysis tools, deletion of 10 nucleotides was observed in* Ty-3* gene sequence for susceptible tomato variety. ACY is a co-dominant indel-based marker that produced clear and strong polymorphic band patterns for resistant plant distinguishing it from its susceptible counterpart. The obtained result correlates with 3:1 segregation ratio of single resistant dominant gene inheritance, which depicted ACY as gene-tag functional marker. This marker is currently in use for screening 968 hybrids varieties and one thousand breeding lines of tomato varieties stocked in Jiangsu Green Port Modern Agriculture Development Company (Green Port). So far, ACY has been used to identify 56 hybrids and 51 breeding lines. These newly detected breeding lines were regarded as potential source of resistance for tomato breeding. This work exploited the sequence of* Ty-3* and subsequently contributed to the development of molecular marker ACY to aid phenotypic selection. We thus recommend this marker to breeders, which is suitable for marker-assisted selection in tomato.

## 1. Introduction

Tomato yellow leaf curl disease (TYLCD) inflicted by whitefly-transmitted species of* Begomovirus* genus (Geminiviridae) is one of the most devastating tomato disease (*S. lycopersicum*) worldwide. The potential of TYLCV to wreak havoc on tomato was initially reported in Israel in the late 1930s, and 1960s, with a severe outbreak on tomato plants recorded in the Mediterranean basin [1]. The biotype B vector* B. tabaci* accounts for the dramatic spread of TYLCV through Africa, Europe, Asia, the Caribbean, and North America [2]. TYLCV is an economically important plant pathogen with yield losses in tomato reaching 100% [1, 3–5]. Tomato yellow leaf curl disease survey followed by sequence analysis across six tomato growing regions in China showed sequence identity of up to 98% among TYLCV isolates [6]. These isolates shared more than 97% sequence identity with TYLCVIL [IL:Reo] (X15656), an Israel isolate [6]. TYLCV with nucleotide sequence homology of more than 90% could be regarded as strains of the same virus [7]. The management of TYLCD is dependent on the intensive use of insecticides to control vector (*B. tabaci*) populations [8]. This chemical control measure is partially effective against insect vectors. Nevertheless, it is expensive and labor intensive and often results in chemical residue buildup, subsequently leading to the development of pesticide resistance insect populations [8–11]. In addition, intensive and inaccurate application of insecticides could generate environmental pollutants and pesticide poisoning via toxin retention in tomato fruit [12]. One of the efficient ways to alleviate or manage TYLCD is introgression of virus resistance into cultivated plants. Previous research works have focused on the determination of natural sources of virus resistance and have been identified in tomato wild relatives including* S. pimpinellifolium*,* S. peruvianum*,* S. chilense*,* S. habrochaites*, and* S. cheesmaniae* [3, 13, 14]. Genetic mapping analysis has led to the identification of different TYLCD resistant/tolerances loci being exploited in tomato breeding from* S. habrochaites* (*Ty-2*),* S. peruvianum* (*ty-5*), and* S. chilense* (*Ty-1*,* Ty-3*, and* Ty-4*) [15–20]. Among these,* S. chilense* has been used extensively as resistance donor parent [21] with* Ty-1* recognized as promising source of TYLCV resistance [22]. The gene* Ty-1 *was identified as allelic to the group of genes* Ty-3*,* Ty-3a,* and* Ty-3b  *[16, 23], whereas, three ORFs were predicted as candidate gene of* Ty-1* /*Ty-3  *[22]. Conventional tomato breeding solely depends on phenotypic traits selection for resistant cultivar. However, the impacts of environmental conditions on phenotype greatly affect its selection efficiency. Field screening is a complex and time consuming process, which requires specific growing conditions [24, 25]. With the advent of marker-assisted selection (MAS) as a modern molecular biology technique, the rapidity and efficiency of resistant cultivar development have been greatly enhanced. Progressive efforts made in the development of* Ty-1*/*Ty-3* resistant genes-derived molecular markers for MAS in tomato, cleaved amplified polymorphic sequence (CAPS), and sequence-characterized amplified region (SCAR) markers are significant [26, 27]. However, concerns have been raised regarding their physical position relative to the resistance gene in the genome, arguing the possibility of false negative or false positive results during breeding programs [4, 26]. To circumvent the aforementioned problem, gene-specific marker technique was introduced, aimed at developing molecular markers with gene specificity [28, 29]. This led to the development of gene-targeted markers (GTMs) and functional markers (FMs), resistance gene based markers (RGMs), and RNA-based markers (RBMs) [30–32]. According to Andersen and Lübberstedt [33] functional markers are polymorphic DNA sequences that are likely to be involved in phenotypic trait variation while gene-targeted markers are gene specific and capable of tagging untranslated regions [34, 35]. RGM can allow tracking the resistant gene in new germplasms, segregating population for promoting gene pyramiding in plant. Based on recently reported research findings, intron length polymorphism seems to be a convenient and reliable source of information with high interspecies transferability [36]. We speculated that marker developed by exploiting such information might contribute to the compensating of the discrepancy observations often encountered between genotypes and phenotypes during breeding programs. Information regarding intron polymorphism based molecular maker for TYLCV resistance marker-assisted selection in tomato breeding is lacking..

Here, we developed and reported a new marker named ACY in the basis of indel-10 nucleotides for* Ty-3*/*Ty-1 *gene* Solyc06g051170 *between cultivated tomato (*S. lycopersicum*) and wild type tomato (*S. pennellii*). This marker can be used as a powerful tool in segregating resistant from susceptible tomato plants. PCR amplification amplicon revealed 132bp and 123bp DNA fragment from TYLCV resistant varieties (R) and susceptible varieties, respectively. Screening of commercial F_1_ hybrids and segregating F_2_ population showed reliability and accuracy with ACY marker.

## 2. Materials and Methods

### 2.1. Plant Materials

Thirty-six (36) commercial varieties, one thousand (1000) breeding lines, and nine hundred and sixty-eight (968) hybrid varieties of tomato were used. The commercial hybrids were purchased from different seed companies. The hybrids and breeding lines constituted the candidate of tomato varieties stock of Jiangsu Green Port Modern Agriculture Development Company (abbreviated as Green Port). Seeding, transplanting, and plants management were handled following the standard practice of Green Port. To conduct the phenotypically selection assay, tomato plants were transplanted in greenhouse to meet the critical period of whiteflies invasion.

### 2.2. Tomato Yellow Leaf Curl Disease Evaluation

A disease rating scales of 0 to 4 were adopted and used as described by Hutton and Scott [37]. On the rating scale, 0, 1, 2, 3, and 4 signified no symptoms, slight symptoms visible upon close inspection, clear symptoms evident on a portion of the plant, heavy symptoms on entire plant, and severe symptoms and stunting of the entire plant, respectively. The plants that were rated on the scale of 0, 1, and 2 were considered resistant, while those of 3 and 4 were susceptible.

### 2.3. DNA Extraction and Polymerase Chain Reaction

DNA was extracted from the fresh leaves of 30- and 60-day-old tomato plants using NuClear Plant Genomic DNA Kit (CWO531M) protocol (CWBiotech, Beijing, China). DNA obtained from 30-day-old plants was used for resistance gene detection while that from 60-day-old plants was used for viral DNA detection. The obtained DNA was adjusted to a final concentration of 10ng/*μ*l. For PCR products to be analyzed by agarose gel, 25*μ*l of PCR reactions mix containing 12.5*μ*l 2xTaq MasterMix (CWBiotech, Beijing, China), 1*μ*l of each forward and reverse primer at 10*μ*M, 1*μ*l of DNA extract, and 9.5*μ*l of sterilized water were used. In the case of polyacrylamide gel analysis, 10*μ*l PCR reactions mix containing 1 *μ*l of DNA extract, 1*μ*l 1 mM dNTPs, 1*μ*l 10xbuffer (-MgCl2), 1*μ*l 25 mM MgCl2, 0.3*μ*l (5 units) Taq DNA polymerase (CWBiotech, Beijing, China), 0.8 *μ*l of each forward and reverse primer at 10*μ*M, and 4.1*μ*l of sterilized water were composed in a PCR tube. Amplification reaction conditions were as follows: initial denaturation of 94° for 2 min, 35 cycles of denaturation at 94° for 30 sec, annealing at 55° for 30 sec, and extension at 72° for 30 seconds followed by 72° for 2 min. The PCR products generated from 25*μ*l reaction mix were separated on 1.5% agarose gel in 0.5x TAE buffer, stained with ethidium bromide, while those obtained from 10 *μ*l reaction mix were analyzed on 8% polyacrylamide gel, stained with silver. Visualization was done under UV and white light, respectively.

### 2.4. Primers Designing and Molecular Screening

The nucleotide sequence of* Ty-3* candidate gene* Solyc06g051170 *was downloaded from ITAG2.4 release genomic annotations database (https://solgenomics.net). The sequence of predicted resistant allele was attributed to wild type tomato* S. pennellii* (accession: HG975518) while susceptible allele was associated with cultivated tomato* S. lycopersicum* (accession: HG975445). Alignment was performed between these two sequences using Basic Local Alignment Search Tool of NCBI database (http://www.ncbi.nlm.nih.gov/). Two indel sequences (9bp and 10bp indels) were observed and exploited for primers designing. Two primer pairs were designed to target these indels sequence. However, only the 10bp indel-based primer showed distingue polymorphic and clear genetic band patterns. The 10bp indel-based primer pair annotated as ACY was used to amplify 132bp DNA fragment from the resistant varieties and 123bp from susceptible varieties. A*Ty-3* linked marker P6-25 was used conjointly for commercial hybrid screening to demonstrate the gene-specific character of ACY. Molecular screening was done following the methods of Yao et al. [38] and Hanson et al. [27] with minor modifications. PCR was performed on three individuals from each plant's varieties using ACY derived primer pair. The entries that showed either 132bp or 123bp length product were considered as homozygote resistant or susceptible plant, respectively, while those that carried both fragments simultaneously were regarded as heterozygote resistant. TYLCV infection was promoted by growing these varieties in an opened door greenhouse under higher pressure of viruliferous whiteflies through July to September, 2017.

### 2.5. Cloning and Sequencing of DNA Fragment

A nested PCR product of ACY-based marker with fragment length of 630bp was obtained using Seq-F and Seq-R primer pair (Table 1) located before ACY-F and after ACY-R primer sequences, respectively. The TYLCV390 primers [3] were used to amplify 390bp DNA fragment from TYLCV in resistant and susceptible plants samples. Cloning was done by ligating these amplicons onto pMD19-T Simple Vector after purification. Constructs (pMD19-T ACY-based marker and pMD19-T-TYLCV fragments) were commercially sequenced by Huada Gene Company, Wuhan. Alignments of obtained sequences were performed using DNAMAN software.

### 2.6. Genetic Analysis of ACY in Segregation of F_2_ Population

A total of 111 F_2_ generations, derived from AG208 (commercial resistant tomato hybrid variety), were grown and used for phenotypic traits evaluation and genotyping with ACY maker. F_2_ population was analyzed according to Yao et al. [38] using Chi-square test with 3:1 segregation ratio (*χ*^2^_0.05_, 1=3.84 as threshold).

## 3. Results

### 3.1. Characterization of Tomato Yellow Leaf Curl Disease

**Figure 1:**
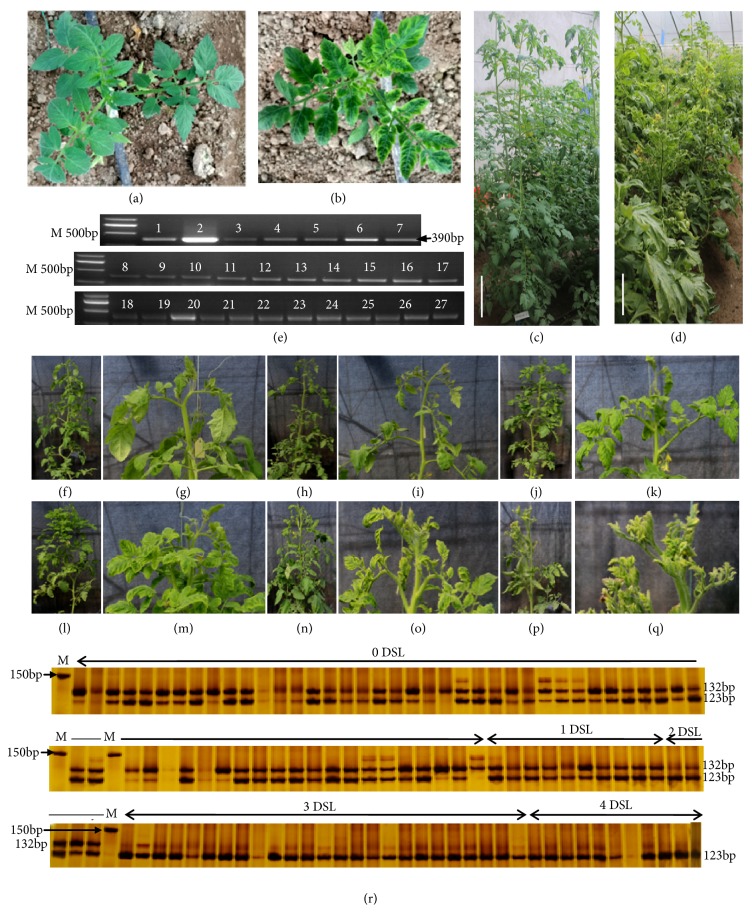
Disease characterization in commercial hybrid varieties and in F2 population derived from commercial resistant AG208 F_1_ hybrid. (a) Five-week-old asymptomatic resistant tomato seedling of AG208 hybrid. (b) Susceptible symptomatic tomato seedling of Hi-tech1. (c) Flowering stage of resistant tomato plants. (d) Susceptible tomato plants. (e) PCR-based diagnostic performed using TYLCY390 primer pair on DNA from tomato plants at flowering stage. (f) Resistant F1 plants form AG208. (g) Upper leaves of resistant F1 plants form AG208. (h-i) Plant upper leaves of F2 individual with 0 DSL. (j-k) Plant upper leaves of F2 individual with 1 DSL. (l-m) Plant upper leaves of F2 individual with 2 DSL. (n-o) Plant upper leaves of F2 individual with 3 DSL. (p-q) Plant upper leaves of F2 individual with 4 DSL. (r) Segregation of ACY marker in F2 population; DSL: disease symptom level, 0: no symptoms, 1: slight symptoms visible upon close inspection, 2: clear symptoms evident on a portion of the plant, 3: heavy symptoms on entire plant, and 4: severe symptoms and stunting of entire plant. The plants belonging to scales 0, 1, and 2 were considered resistant, while 3 and 4 were susceptible. Resistance fragment = 132bp and susceptible fragment = 123bp.

Phenotypic characterization of commercial tomato plants against TYLCV showed consistency in phenotype between greenhouse screening evaluation result and that provided by the respective seed supplier companies. In fact, twenty-five (25) hybrid varieties were categorized as resistant to TYLCV and eleven (11) as susceptible to TYLCV infection following their field performance (Suppl.  Table S1 and Figures 1(a)-1(d)). PCR-based diagnostic performed using a primer pair derived from TYLCV DNA sequence (TYLCV390 primer) as described by Kil et al. [3] yielded DNA fragment of 390bp from all the infected plants (Figure 1(e)). The obtained sequences from the cloned 360bp amplicon in alignment with that of different TYLCV isolate from Israel and Japan revealed 98% sequence homology, especially with TYLCV-Israel strain [IR:Boj:28-2] (Figure S.1). This is in conformity with previously reported Chinese TYLCV isolates sharing more than 97% nucleotide sequence identity with TYLCVIL [IL:Reo] (X15656) [6].

### 3.2. Segregation Analysis with ACY Marker

The ACY marker was validated for segregating F_2_ population derived from AG208, one of the control commercial hybrids carrying* Ty-3 *gene. Phenotypic evaluation of 111 genotypes of F_2_ population based on disease symptom showed 75 resistant and 36 susceptible plants. This is equivalent to the 3:1 segregation ratio revealed by Chi-square test (*χ*^2^=3.26 < 3.84 of *χ*^2^_0.05_ with df = 1) (Table S2). Sixty plants were found with no symptoms, ten with slight symptoms expression, and five (5) with visible symptoms; twenty-five showed severe symptoms on entire plant, and eleven (11) showed severe symptoms and stunting. The first three groups suit the rating scales of 0, 1, and 2, respectively, and were considered resistant while the two last groups were considered susceptible (belonging to rating scales of 3 and 4) (Figures 1(f)-1(q)). These match the results obtained when ACY marker was used for genotyping (Figure 1(r)). These results support the assumption that resistance character is actually under control of a single dominant resistant gene.

### 3.3. Validation and Accuracy of ACY Marker

**Figure 2:**
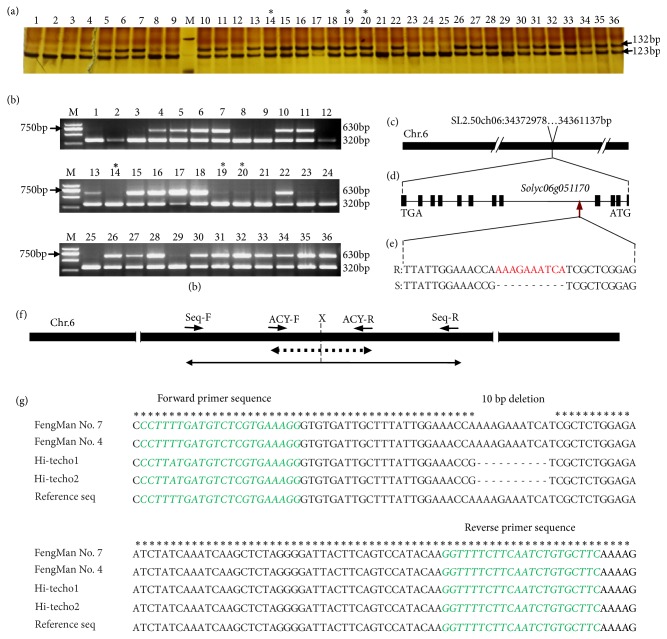
Genetic characterization of ACY marker. (a) Polyacrylamide gel and (b) agarose gel electrophoresis of 36 commercial tomato hybrids using a newly developed ACY marker and* Ty-3* linked marker P6-25, respectively. (c) Predicted position of ACY on tomato chromosome 6. (d) Structure of* Ty-3* gene with 12 exons and 11 introns. (e) 10bp indel predicted between* S. pennellii *and* S. lycopersicum*; ATG and TGA represent start codon and stop codon, respectively; R indicates* S. pennellii *allele and S indicates* S. lycopersicum*. (f) Graphical representation of fragment size and primers position for ACY marker and those of DNA sequencing; X and vertical line indicate indel position; dashed line with double arrows indicates ACY amplified products (132bp and 123 bp); and double arrows line shows amplified DNA fragment for sequencing (630bp). (g) DNA sequencing results confirming 10bp insertion in resistant genotype and 10bp deletion in susceptible one; nucleotides in Italic are forward and reverse primers sequence from ACY. M in (a) and (b) were 500bp and 2000bp DNA Ladder Marker, respectively. 1: Pinkebabe, 2: Qianxi, 3: Huangrong, 4: Jiaxina (74-112), 5: Manxina (73-47) Messina, 6: Futesi 72-152, 7: Mantian 2025, 8: Hi-tech1, 9: Hi-tech2, 10: YuyiliangJingjing, 11: Aomei No. 1, 12: Oudun, 13: AG112, 14: AG115, 15: AG158, 16: AG1330, 17: Fengman No. 7, 18: Fengman No. 4, 19: Dongfeng 601, 20: NongboFenba No 1510, 21: ZhonghuaLvbao, 22: Jinfan102, 23: Duoxi13-1, 24: Nongqing 12-7, 25: Jinpeng 703, 26: Tianbao 326, 27: Yabao, 28: Luola, 29: Beiying, 30: Qidali, 31: Fenshou (74-560) RZ F1, 32: AG208, 33: Mantian 2218, 34: Mantian 2199, 35: Jingfan 502 (*Ty1*,* Ty3a*), 36: Hongshuang Xi (4224; Asterisk (*∗*) represents the recombinants varieties.

The developed ACY marker has potentials in differentiating resistant and susceptible tomato varieties to TYLCD. Thirty-six (36) tomato hybrids were screened using ACY, for commercial resistant and susceptible hybrid varieties in order to check the marker's accuracy and reliability. Molecular assay using ACY marker revealed twenty-four commercial resistant varieties with heterozygote allele (123bp/132bp), one resistant hybrid with homozygote resistant allele (132bp), and eleven (11) susceptible varieties with homozygous susceptible allele (123bp) (Figure 2(a)). This result is in agreement with the field performance data of the commercial hybrids. In order to confirm ACY for being a resistance gene-specific marker, which is located within the* Ty-3 *gene itself, a linked maker P6-25 commonly used for marker-assisted selection for* Ty-3* gene was later involved in genotyping commercial tomato hybrids. Genotype of the thirty-six (36) commercial varieties using P6-25 revealed fourteen with homozygote susceptible allele (320bp) and twenty-two with heterozygote resistant allele (320bp/630bp) (Figure 2(b)). It is worthwhile to note that 320bp amplicon for P6-25 marker is tagged to susceptibility while the amplicons 540bp (*Ty-3*), 630bp (*Ty-3a*), or 660bp (*Ty-3b*) were for resistivity, suggesting that the resistance commercial hybrid might harbor* Ty-3a* gene. In this regard, all the commercial hybrids might carry* Ty-3a* gene. Genotypic differences were recorded with AG115, NongboFenba 1510, and Dongfeng 601 hybrids differing from the phenotypic observation. Although genotypic screening with P6-25 revealed that these three hybrids possess the marker locus at the homozygous susceptible state, their phenotypic expression was quiet different. Their physical appearance showed resistance against TYLCV, while molecular analysis data showed susceptibility, bringing about discrepancy between phenotypic and molecular analysis. This difference might be a result of crossing-over between the P6-25 marker and* Ty-3* locus; thus, the three hybrids can be considered as recombinant varieties. Prediction using Blast tools in Gramene database (http://www.gramene.org) has located P6-25 to approximately 620kb from the* Ty-3* gene locus, while ACY was found in fourth intron of the* Ty-3* gene* Solyc06g051170* (Figures 2(c)-2(d)). The developed ACY marker is an efficient tool that can help in correcting the differences between genotype and phenotype analysis of tomato plants. Functional characteristic and efficiency evaluation of ACY marker through sequence analysis using Seq primers showed 10bp insertion in wild type gene possessed by resistant varieties, not found in susceptible varieties (Figure 2(e)). Graphical representations of ACY and Seq primer amplified bands are highlighted in Figure 2(f). The sequencing result has confirmed 10 nucleotides deletion in susceptible varieties as compared to resistant varieties (Figure 2(g)).

### 3.4. Selection of the Candidate Hybrid Varieties and Breeding Lines of Green Port Company for* Ty-3* Resistance Gene

Phenotypic traits evaluation and molecular screening test using ACY marker together with P6-25 were performed on one thousand breeding lines and 968 hybrids stocked in Green Port Company (Suppl.  Figure S1 a, b; Table S3 and Table S4). Among the breeding lines, 51 showed 132bp of PCR amplicon, which revealed the presence of* Ty-3* resistance gene using ACY. In order to investigate whether there is introgression of three different alleles (*Ty-3*,* Ty-3a*, and* Ty-3b*) of* Ty-3 *resistance gene as well as* Ty-1 *in these breeding lines, P6-25 and SCAR1 were, respectively, used for screening the resistant breeding lines. Twenty-five out of 51 resistant breeding lines were detected with* Ty-3a*, 24 lines with* Ty-3*, and two with* Ty-1 *(Suppl. Table S3 and Suppl.  Figure S1c-d). No line was detected to harbor* Ty-3b* allele. Meanwhile, molecular screening was done using SCAR2 (*Ty-2*) to determine whether or not there is another source of resistance gene in these lines (Suppl.  Figure S1e). It was found that breeding lines Y-172 and Y-49 possess* Ty-2* (Suppl. Table S3). Another thirty-five lines also expressed disease resistance in the field. However,* Ty-1*,* Ty-3*, and* Ty-2* resistance genes could not be detected. It could be proposed that there might be an alternative source of resistance against TYLCD for these varieties. In case of the 968 candidate hybrids molecular screening, 56 carry* Ty-3* resistance gene as revealed by molecular markers ACY. This result is further confirmed using the* Ty-3* linked marker P6-25 for screening. According to the result obtained from P6-25, we speculate that all the selected 56 candidate hybrids carry* Ty-3a* resistance gene (Suppl. Table S4).

## 4. Discussion

Numerous molecular markers associated with important genes for plants of agricultural and horticultural interests have been developed [36]. Introgression of resistance gene into cultivated variety has been the principal route of breeding against tomato yellow leaf disease (TYLCD); this process requires development of molecular marker for resistance gene tracking. Nowadays, resistance gene based techniques have the advantage of creating molecular markers linked to potential functional genes, with the ability of providing information of the targeted gene [39]. The major TYLCD associated markers generated so far include a tightly linked molecular marker SCAR1 for screening of* Ty-1* [40], P6-25 and FLUW25 for screening of* Ty-3*,* Ty-3a*, and* Ty-3b* [13, 23], SCAR2 and P1-16 for* Ty-2* identification [13, 26]. Most of these markers are recognized as arbitrarily amplified DNA (AAD) fragment markers [36]. There is little or limited information about the genetic distance between these markers and resistance genes locus. In most cases a simple recombination event is sufficient to negate their utility, thus limiting their use in MAS [41]. As an example, genomic location of P6-25 could cause recombination event between marker and gene of interest as demonstrated in this study. Nowadays, gene-targeted and functional marker technologies have constituted a breakthrough in marker-assisted selection. In this study, we developed a new marker named “ACY” for use in MAS based on intron polymorphism of* Ty-3* resistance gene in tomato. Insertion-deletions (indels) of introns are becoming important genetic markers for many plant taxa [42]. Here, ACY marker type could be attributed to gene-targeted and functional markers group (GTMs and FMs) [36], especially the intron-targeting polymorphism (ITP) marker as classified by Weining and Langridge [43]. Interestingly, recombination event is rare or impossible between ACY and* Ty-3.*

The* Ty-3 *is one of the most promising resistant genes commonly used against the occurrence of TYLCD in China. Upon the fine mapping experimental research, three ORFs,* Solyc06g051170*,* Solyc06g051180*, and* Solyc06g051190,* were predicted as candidate genes [22]. Much indel information has been recorded from the alignment of* Ty-3* DNA sequence of different cultivated and wild type tomato varieties. Several indel markers have been designed for polymorphism assay using resistant and susceptible varieties. The ACY is co-dominant marker that could efficiently distinguish TYLCV resistant allele from susceptible counterpart based on 10 nucleotides difference. It was observed in this experiment that ACY marker is located in the vicinity of the exon close to the biggest intron section of* Solyc06g051170 *locus. It is accurately validated in major resistant commercial hybrids used in this experiment, with most of them carrying* Ty-3* resistant gene. Similarly, its segregation ratio was observed in AG208 derived F_2_ population which completely matched a single resistant gene. Virus strain diagnostic test from these commercial hybrids (including the resistant and susceptible hybrids) showed more than 98% sequence homology with previously reported TYLCV isolates from China, Israel TYLCV-IL, and Japan TYLCV-Tosa; this corroborated the findings of Wu et al. [44] and Zhang et al. [6]. Therefore, it could be inferred that all or most of the plants could be infected naturally, thus making the field phenotypic observation reliable. Thus, ACY marker could be recommended to breeders for detecting and tracking of* Ty-3* in tomato resistance breeding program against TYLCV and will significantly improve tomatoes production. It may not be advisable to depend solely on a single type of molecular marker for MAS because it may not always generate accurate band pattern under different experimental conditions. A diversity of molecular markers could be effective for sustainability and validation of MAS processes.

This ACY marker is being used for screening tomato varieties comprised of 968 hybrid varieties and one thousand breeding lines stocked in Green Port Company for breeding program. Actually, we have selected 56 hybrids varieties with* Ty-3a* and 51 breeding lines that carry different alleles of* Ty-3* gene, including* Ty-3*,* Ty-3a,* and* Ty-1,* based on P6-25 and SCAR1 markers, respectively. Diagnostic PCR using ACY has shown 123bp/132bp or 132bp amplicons from all the selected varieties, indicating that they incorporate* Ty-3* resistance gene. Here we speculate that ACY marker may have a broad-spectrum application with ability to simultaneously detect the presence of* Ty-3*,* Ty-3a*, and* Ty-1* resistant alleles. However, additional research with ACY marker amplifying* Ty-3 *resistant gene form the genotypes carrying* Ty-3*,* Ty-3a*, and* Ty-1* allele would be required in future for confirmation. The breeding lines that manifest disease resistance without carrying* Ty-3 *are considered having other source of resistance genes (*Ty-4*,* Ty-5*, and* Ty-6*). These lines can be subjected to further molecular screening study using the available molecular markers for those resistance genes. The newly selected hybrid varieties could have resistance potential and implication in tomatoes production and in agricultural extension work in China. The newly identified breeding lines constitute promising breeding materials, which could be used in resistance breeding as new sources of* Ty-3* resistance for tomatoes breeding program. We believe that the newly developed marker could serve as an alternative breeding tool to immensely assist the breeders to tract* Ty-3* allele in tomato plants for breeding program. Most of the* Ty* resistance genes have been reported to be derived from different wild tomato species such as* Solanum chilense*,* Solanum peruvianum*,* Solanum hirsutum*,* Solanum pimpinellifolium*, and* Solanum cheesmanii*; however, little TYLCV resistance breeding research work involving* Solanum pennellii *has been done [13, 16, 45]. Here, ACY specific marker is developed based on the predicted genomic sequence of* Ty-3* from* S. pennellii*, indicating that this wild species may also contain TYLCD resistance gene. Up to date,* Ty-3* is commonly known to be derived from* S. chilense*, and this is the first report suspecting* S. pennellii* to harbor* Ty-3*. Besides the development of new marker for the promotion of breeding efficiency, this study tends also to bring closer two tomato wild species in terms of resistance to TYLCD. This information provides evidence of using* S. pennellii* in resistant tomato breeding program.

## Figures and Tables

**Table 1:** The primer sequences used in amplifying targeted fragments.

Marker name	Forward primer sequence (5′-3′)	Reverse primer sequence (3′-5′)
ACY*∗*	CCTTATGATGTCTCGTGAAAGG	GAAGCACAGATTGAAGAAAACC
Seq	ATACTTTTCTCGTGCCTTCTC	AGCTTATTTTGCTGGCTCATA
TYLCV390	GATGGCCGCGCCTTTTCCTTTTATGTGG	GCTGCTGTATGGGCTGTCGAAGTTCAG
P6-25	GGTAGTGGAAATGATGCTGCTC	GCTCTGCCTATTGTCCCATATATAACC
SCAR1	CAATTTATAGGTGTTTTTGGGACATC	GTTCAACACTTGGCCAATGCTTACG
SCAR2	TGGCTCATCCTGAAGCTGATAGCGC	AGTGTACATCCTTGCCATTGACT

*∗* Primer sequence of the newly developed ACY marker

## Data Availability

1. The primers sequence and molecular screening data used to support the findings of this study are included within the article. 2. The patent of the newly developed ACY marker is under submission; however, the primer sequence data is available here for the scientific community. 3. Previously reported markers data and the sequence of their corresponding primers used to support this study are available in this article (Table 1). The related prior studies are cited in References. The field screening of the breeding lines and all the hybrids varieties (Our own developed hybrid varieties and the purchased commercial hybrid varieties) as well as their respective molecular screening data used to support the findings of this study are included within the Supplementary Materials. 5. The tomato breeding lines used to support the findings of this study were supplied by Professor Si Longting, Manager of Green Port Company Research Center, and then they are under license and cannot be made freely available. Requests for access to these materials should be made to the Manager of Research Center of Green Port Company Professor Si Longting, mobile phone: 0086 188 0060 2136, e-mail: 2582259972@qq.com, or to Dr. Adedze YM Nevame, Senior Scientist in Green Port Company, e-mail: amen.nevame07@yahoo.fr 6. All the tomato hybrid varieties used to support the findings of this study are currently under commercialization. Requests for them will need 12 months after publication of this article.

## Supplementary Materials

Supplementary Materials Table S1: phenotypic and molecular screening results based on 36 F1 generation seeds of commercial tomato hybrid varieties. Table S2: evaluation of F2 generation of hybrid stock for resistance for tomato yellow leaf curl virus. Table S3: tomato breeding lines phenotypic and genotypic selection assay for two consecutive periods. Table S4: hybrid varieties selected during the period of two consecutive phenotypic observation and molecular analysis assays. Figure S1: comparison of the 390bp DNA sequences between six different strains of TYLCV: Ty-IS=TYLCV-Israel strain [IR:Boj:28-2], Ty-Mld=TYLCV-Isolate Mld, Ty-Gs=TYLCV-Isolate Goseong Ty-MMN=TYLCV-Isolate Mu_Mu5-N, Ty-JT= TYLCV-Israel[Japan:Tosa:2005], Ty-GP= TYLCV-Green Port. Figure S2: molecular screening results of the hybrid varieties and breeding lines; (a) molecular screening of breeding lines (R-lines=resistant lines and S-lines= susceptible lines) and hybrid varieties using ACY, where 132bp indicates the presence of resistance genes either* Ty-1*,*Ty-3 *or* Ty-3a *and 123bp indicates the presence of susceptible gene* ty-1*,* ty-3*, or* ty-3*; (b) molecular screening of hybrid varieties using P6-25, where 630bp indicates the presence of resistance gene* Ty-3a *and 320bp the presence of susceptible gene* ty-3*; (c) molecular screening of breeding lines using P6-25, where 630bp indicates the presence of resistance gene* Ty-3a*, 450bp the presence of resistance gene* Ty-3*, and 320bp the presence of susceptible gene* ty-3*; (d) molecular screening of breeding lines using SCAR1, where 530bp indicates presence of resistance gene* Ty-1 *and 610bp the presence of susceptible gene* ty-1*; (e) molecular screening of breeding lines using SCAR2, where 900bp indicates the presence of resistance gene* Ty-2 *and 800bp the presence of susceptible one* ty-2*; M= marker.
