# Supplementary material for: Development of a New Molecular Marker for the Resistance to Tomato Yellow Leaf Curl Virus
Source: Biomed Res Int. 2018 Jul 17;2018:8120281. doi: 10.1155/2018/8120281 (PMC6076955; doi:10.1155/2018/8120281)
Supplement: Supplementary Materials — Table S1: phenotypic and molecular screening results based on 36 F1 generation seeds of commercial tomato hybrid varieties. Table S2: evaluation of F2 generation of hybrid stock for resistance for tomato yellow leaf curl virus. Table S3: tomato breeding lines phenotypic and genotypic selection assay for two consecutive periods. Table S4: hybrid varieties selected during the period of two consecutive phenotypic observation and molecular analysis assays. Figure S1: comparison of the 390bp DNA sequences between six different strains of TYLCV: Ty-IS=TYLCV-Israel strain [IR:Boj:28-2], Ty-Mld=TYLCV-Isolate Mld, Ty-Gs=TYLCV-Isolate Goseong Ty-MMN=TYLCV-Isolate Mu_Mu5-N, Ty-JT= TYLCV-Israel[Japan:Tosa:2005], Ty-GP= TYLCV-Green Port. Figure S2: molecular screening results of the hybrid varieties and breeding lines; (a) molecular screening of breeding lines (R-lines=resistant lines and S-lines= susceptible lines) and hybrid varieties using ACY, where 132bp indicates the presence of resistance genes either Ty-1,Ty-3 or Ty-3a and 123bp indicates the presence of susceptible gene ty-1, ty-3, or ty-3; (b) molecular screening of hybrid varieties using P6-25, where 630bp indicates the presence of resistance gene Ty-3a and 320bp the presence of susceptible gene ty-3; (c) molecular screening of breeding lines using P6-25, where 630bp indicates the presence of resistance gene Ty-3a, 450bp the presence of resistance gene Ty-3, and 320bp the presence of susceptible gene ty-3; (d) molecular screening of breeding lines using SCAR1, where 530bp indicates presence of resistance gene Ty-1 and 610bp the presence of susceptible gene ty-1; (e) molecular screening of breeding lines using SCAR2, where 900bp indicates the presence of resistance gene Ty-2 and 800bp the presence of susceptible one ty-2; M= marker. [file 8120281.f1.pdf]

Table S1. Phenotypic and molecular screening results based on 36 F1 generation seeds of commercial tomato hybrid varieties.

Serial No	Variety Name	Type of seed	Field screening P2017.04.9	Molecular Screening		
				P6-252017.4	ACY2017.4	ACY2017.9
1	Pinkebabe	F1	S	*ty-3a/ty-3a*	*ty-3/ty-3*	*ty-3/ty-3*
2	Qianxi	F1	S	*ty-3a/ty-3a*	*ty-3/ty-3*	*ty-3/ty-3*
3	Huangrong	F1	S	*ty-3a/ty-3a*	*ty-3/ty-3*	*ty-3/ty-3*
4	Jiaxina（74-112）	F1	R	*TY-3a/ty-3a*	*TY-3/ty-3*	*TY-3/ty-3*
5	Manxina（73-47）Messina	F1	S	*ty-3a/ty-3a*	*ty-3/ty-3*	*ty-3/ty-3*
6	Futesi 72-152	F1	R	*TY-3a/ty-3a*	*TY-3/ty-3*	*TY-3/ty-3*
7	Mantian2025	F1	R	*TY-3a/ty-3a*	*TY-3/ty-3*	*TY-3/ty-3*
8	Hi-tech1	F1	S	*ty-3a/ty-3a*	*ty-3/ty-3*	*ty-3/ty-3*
9	Hi-tech2	F1	S	*ty-3a/ty-3a*	*ty-3/ty-3*	*ty-3/ty-3*
10	YuyiliangJingjing	F1	R	*TY-3a/ty-3a*	*TY-3/ty-3*	*TY-3/ty-3*
11	Aomei No1	F1	R	*TY-3a/ty-3a*	*TY-3/ty-3*	*TY-3/ty-3*
12	Oudun	F1	**S**	*ty-3a/ty-3a*	*ty-3/ty-3*	*ty-3/ty-3*
13	AG112	F1	R	*ty-3a/ty-3a*	*TY-3/ty-3*	*TY-3/ty-3*
14	AG115*	F1	R	*ty-3a/ty-3a*	*TY-3/ty-3*	*TY-3/ty-3*
15	AG158	F1	R	*TY-3a/ty-3a*	*TY-3/ty-3*	*TY-3/ty-3*
16	AG1330	F1	R	*TY-3a/ty-3a*	*TY-3/ty-3*	*TY-3/ty-3*
17	FengmanNo7	F1	R	*TY-3a/ty-3a*	*TY-3/ty-3*	*TY-3/ty-3*
18	FengmanNo4	F1	R	*TY-3a/ty-3a*	*TY-3/ty-3*	*TY-3/ty-3*
19	Dongfeng 601*	F1	R	*ty-3a/ty-3a*	*TY-3/ty-3*	*TY-3/ty-3*
20	NongboFenba No 1510*	F1	R	*ty-3a/ty-3a*	*TY-3/ty-3*	*TY-3/ty-3*
21	ZhonghuaLvbao	F1	S	*ty-3a/ty-3a*	*ty-3/ty-3*	*ty-3/ty-3*
22	Jinfan102 (*Ty-1*,*Ty-3*)[a]	F1	R	*TY-3a/ty-3a*	*TY-3/ty-3*	*TY-3/ty-3*
23	Duoxi13-1	F1	S	*ty-3a/ty-3a*	*ty-3/ty-3*	*ty-3/ty-3*
24	Nongqing 12-7	F1	S	*ty-3a/ty-3a*	*ty-3/ty-3*	*ty-3/ty-3*
25	Jinpeng703	F1	S	*ty-3a/ty-3a*	*ty-3/ty-3*	*ty-3/ty-3*
26	Tianbao 326	F1	R	*TY-3a/ty-3a*	*TY-3/ty-3*	*TY-3/ty-3*
27	Yabao	F1	R	*TY-3a/ty-3a*	*TY-3/ty-3*	*TY-3/ty-3*
28	Luola	F1	R	*TY-3a/ty-3a*	*TY-3/ty-3*	*TY-3/ty-3*
29	Beiying	F1	**S**	*ty-3a/ty-3a*	*ty-3/ty-3*	*ty-3/ty-3*
30	Qidali	F1	R	*TY-3a/ty-3a*	*TY-3/ty-3*	*TY-3/ty-3*
31	Fenshou（74-560） RZ F1	F1	R	*TY-3a/ty-3a*	*TY-3/ty-3*	*TY-3/ty-3*
32	AG208 (*Ty-3*)[a]	F1	R	*TY-3a/ty-3*	*TY-3/ty-3*	*TY-3/ty-3*
33	Mantian 2218	F1	R	*TY-3a/ty-3a*	*TY-3/ty-3*	*TY-3/ty-3*
34	Mantian 2199	F1	R	*TY-3a/ty-3a*	*TY-3/ty-3*	*TY-3/ty-3*
35	Jingfan 502 (*Ty-1*,*Ty-3*)[a]	F1	R	*TY-3a/ty-3a*	*TY-3/ty-3*	*TY-3/ty-3*
36	Hongshuangxi（4224）	F1	R	*TY-3a/ty-3a*	*TY-3/ty-3*	*TY-3/ty-3*

P2017.4.9= Phenotypic evaluation in April and September 2017, R=resistant, S=susceptible, F1= hybrid variety, P6-252017.4= Linked marker P6-25 used for molecular screening in April 2017，

ACY2017.4= ACY marker used for molecular screening in April 2017，ACY2017.9= ACY marker used for molecular screening in September 2017. * indicates recombinant lines, a represents the resistance gene indicated by seed supplier companies.

Description: Field phenotying and molecular screening of 36 commercial tomatovarieties purchased from different seed companies in China. The tomato yellow leaf curl disease susceptibility of each commercial hybrid was indicated by the seed suppliers. Here, we have re-conducted the phenotying experiment and found a perfect agreement between the supplier's indications and our field results. Further, molecular screening of these hybrids using P6-25 has detected Ty-3 resistance gene. Interestingly, two consecutive molecular screening of these hybrids using new marker ACY has corroborated the field phenotying result (TableS1)

Table S2. Evaluation of F2 generation of hybrid stock for resistance for tomato yellow leaf curl virus.

Type of test	F2 population			$\chi 2$value
	R	S	Total	
Field test	75	36	111	3.26<3.84 ($\chi 2_{0.05}$,df=1)
Molecular test	75	36	111	3.26<3.84 ($\chi 2_{0.05}$,df=1)

R-resistant plants; S-susceptible plants; Chi-square value indicated that the ratio of resistance- to-susceptible F2 populations matched with the expected ratio (3:1) as 3.26 < 3.84 ($\chi 2_{0.05}$, df =1).

Table S3: Tomato breeding lines phenotypic and genotypic selection assay for two consecutive periods.

Code	Field phenotyping		ACY		P6-25	SCAR2	SCAR1
	P2017.4	P2017.9	M2017.4	M2017.9	M2017.9	M2017.9	M2017.9
T-003	R	R	Ty-3/Ty-3	Ty-3/Ty-3	Ty-3/Ty-3	ty-2/ty-2	ty-1/ty-1
T-027	R	R	Ty-3/Ty-3	Ty-3/Ty-3	Ty-3/Ty-3	ty-2/ty-2	ty-1/ty-1
T-033	R	R	Ty-3/Ty-3	Ty-3/Ty-3	Ty-3/Ty-3	ty-2/ty-2	ty-1/ty-1
T-034	R	R	Ty-3/Ty-3	Ty-3/Ty-3	Ty-3a/Ty-3a	ty-2/ty-2	ty-1/ty-1
T-035	R	R	Ty-3/Ty-3	Ty-3/Ty-3	Ty-3/Ty-3	ty-2/ty-2	ty-1/ty-1
T-039	R	R	Ty-3/ty-3	Ty-3/ty-3	Ty-3a/ty-3	ty-2/ty-2	ty-1/ty-1
T-040	R	R	N/A	Ty-3/ty-3	Ty-3/ty-3	ty-2/ty-2	ty-1/ty-1
T-048	R	R	Ty-3/ty-3	Ty-3a/ty-3a	Ty-3/ty-3	ty-2/ty-2	ty-1/ty-1
T-068	R	R	Ty-3/ty-3	Ty-3/Ty-3	Ty-3/Ty-3	ty-2/ty-2	ty-1/ty-1
T-070	R	R	Ty-3/ty-3	Ty-3/ty-3	Ty-3/ty-3	ty-2/ty-2	ty-1/ty-1
T-076	R	R	Ty-3/ty-3	Ty-3/ty-3	Ty-3/ty-3	ty-2/ty-2	ty-1/ty-1
T-080	R	R	Ty-3/ty-3	Ty-3/ty-3	Ty-3/ty-3	ty-2/ty-2	ty-1/ty-1
T-086	R	R	Ty-3/ty-3	Ty-3/ty-3	Ty-3/ty-3	ty-2/ty-2	ty-1/ty-1
T-090	R	R	Ty-3/ty-3	Ty-3/ty-3	Ty-3/ty-3	ty-2/ty-2	ty-1/ty-1
T-120	R	R	N/A	Ty-3/Ty-3	Ty-3/Ty-3	ty-2/ty-2	ty-1/ty-1
T-126	R	R	N/A	Ty-3/Ty-3	Ty-3a/Ty-3a	ty-2/ty-2	ty-1/ty-1
T-132	R	R	Ty-3/ty-3	Ty-3/Ty-3	Ty-3/Ty-3	ty-2/ty-2	ty-1/ty-1
T-138	R	R	Ty-3/ty-3	ty-3/ty3	ty-3/ty3	ty-2/ty-2	ty-1/ty-1
T-153	R	R	N/A	Ty-3/Ty-3	Ty-3/Ty-3	ty-2/ty-2	ty-1/ty-1
T-162	R	R	N/A	Ty-3/Ty-3	Ty-3/Ty-3	ty-2/ty-2	ty-1/ty-1
T-164	R	R	N/A	Ty-3/Ty-3	Ty-3a/Ty-3a	ty-2/ty-2	ty-1/ty-1
T-166	R	R	Ty-3/ty-3	Ty-3/Ty-3	Ty-3/Ty-3	ty-2/ty-2	ty-1/ty-1
T-169	R	R	N/A	Ty-3/Ty-3	Ty-3a/Ty-3a	ty-2/ty-2	ty-1/ty-1
T-176*	R	R	N/A	Ty-3/Ty-3	ty-3/ty-3	ty-2/ty-2	ty-1/ty-1
T-181	R	R	Ty-3/Ty-3	Ty-3/Ty-3	Ty-3/Ty-3	ty-2/ty-2	ty-1/ty-1
T-185*	R	R	N/A	Ty-3/Ty-3	ty-3/ty-3	ty-2/ty-2	ty-1/ty-1
T-188	R	R	N/A	Ty-3/Ty-3	Ty-3/Ty-3	ty-2/ty-2	ty-1/ty-1
T-202	R	R	N/A	Ty-3/Ty-3	Ty-3a/Ty-3a	ty-2/ty-2	ty-1/ty-1
T-252	R	R	N/A	Ty-3/Ty-3	Ty-3a/Ty-3a	ty-2/ty-2	ty-1/ty-1
T-257	R	R	N/A	ty-3/ty-3	ty-3/ty-3	ty-2/ty-2	ty-1/ty-1
L-2	R	R	N/A	ty-3/ty-3	ty-3/ty-3	ty-2/ty-2	ty-1/ty-1
L-5	R	R	N/A	Ty-3/Ty-3	Ty-3/Ty-3	ty-2/ty-2	ty-1/ty-1
L-7	R	R	N/A	Ty-3/Ty-3	Ty-3a/Ty-3a	ty-2/ty-2	ty-1/ty-1
L-25	R	R	N/A	Ty-3/Ty-3	Ty-3a/Ty-3a	ty-2/ty-2	ty-1/ty-1
L-32*	R	R	N/A	Ty-3/Ty-3	ty-3/ty-3	ty-2/ty-2	ty-1/ty-1
L-38	R	R	N/A	Ty-3/Ty-3	Ty-3a/Ty-3a	ty-2/ty-2	ty-1/ty-1
L-110*	R	R	N/A	Ty-3/Ty-3	ty-3/ty-3	ty-2/ty-2	ty-1/ty-1
L-120	R	R	N/A	Ty-3/Ty-3	Ty-3a/Ty-3a	ty-2/ty-2	ty-1/ty-1
L-132	R	R	N/A	Ty-3/Ty-3	Ty-3/Ty-3	ty-2/ty-2	ty-1/ty-1
L-156	R	R	Ty-3/ty-3	Ty-3/Ty-3	Ty-3/Ty-3	ty-2/ty-2	ty-1/ty-1

L-163	R	R	N/A	Ty-3/Ty-3	Ty-3a/Ty-3a	ty-2/ty-2	ty-1/ty-1
L-172	R	R	N/A	Ty-3/Ty-3	Ty-3a/Ty-3a	ty-2/ty-2	ty-1/ty-1
L-188	R	R	N/A	Ty-3/Ty-3	Ty-3/Ty-3	ty-2/ty-2	ty-1/ty-1
L-205	R	R	N/A	ty-3/ty-3	ty-3/ty-3	ty-2/ty-2	ty-1/ty-1
L-222	R	R	N/A	ty-3/ty-3	ty-3/ty-3	ty-2/ty-2	ty-1/ty-1
L-230	R	R	N/A	Ty-3/Ty-3	Ty-3a/Ty-3a	ty-2/ty-2	ty-1/ty-1
L-251	R	R	Ty-3/ty-3	ty-3/ty-3	ty-3/ty-3	ty-2/ty-2	ty-1/ty-1
L-314	R	R	Ty-3/Ty-3	Ty-3/Ty-3	Ty-3a/Ty-3a	ty-2/ty-2	ty-1/ty-1
L-324	R	R	Ty-3/Ty-3	Ty-3/Ty-3	Ty-3a/Ty-3a	ty-2/ty-2	ty-1/ty-1
Y-7	R	R	N/A	ty-3/ty-3	ty-3/ty-3	ty-2/ty-2	ty-1/ty-1
Y-16	R	R	Ty-3/ty-3	ty-3/ty-3	ty-3/ty-3	ty-2/ty-2	ty-1/ty-1
Y-19	R	R	N/A	ty-3/ty-3	ty-3/ty-3	ty-2/ty-2	ty-1/ty-1
Y-20	R	R	N/A	Ty-3/Ty-3	Ty-3/Ty-3	ty-2/ty-2	ty-1/ty-1
Y-26	R	R	N/A	ty-3/ty-3	ty-3/ty-3	ty-2/ty-2	ty-1/ty-1
Y-40	R	R	Ty-3/Ty-3	Ty-3/Ty-3	Ty-3/Ty-3	ty-2/ty-2	ty-1/ty-1
Y-49	R	R	N/A	ty-3/ty-3	ty-3/ty-3	Ty-2/Ty-2	ty-1/ty-1
Y-60	R	R	N/A	ty-3/ty-3	ty-3/ty-3	ty-2/ty-2	ty-1/ty-1
Y-77	R	R	Ty-3/ty-3	Ty-3a/ty-3	Ty-3/ty-3	ty-2/ty-2	ty-1/ty-1
Y-89	R	R	Ty-3/Ty-3	ty-3/ty-3	ty-3/ty-3	ty-2/ty-2	ty-1/ty-1
Y-90	R	R	Ty-3/ty-3	ty-3/ty-3	ty-3/ty-3	ty-2/ty-2	ty-1/ty-1
Y-96	R	R	N/A	ty-3/ty-3	ty-3/ty-3	ty-2/ty-2	ty-1/ty-1
Y-113	R	R	N/A	ty-3/ty-3	ty-3/ty-3	ty-2/ty-2	ty-1/ty-1
Y-121	R	R	N/A	Ty-/Ty-3	Ty-3/Ty-3	ty-2/ty-2	ty-1/ty-1
Y-125	R	R	Ty-3/Ty-3	Ty-3/Ty-3	Ty-3/Ty-3	ty-2/ty-2	ty-1/ty-1
Y-127	R	R	Ty-3/ty-3	Ty-3/ty-3	Ty-3/ty-3	ty-2/ty-2	ty-1/ty-1
Y-133	R	R	N/A	ty-3/ty-3	ty-3/ty-3	ty-2/ty-2	ty-1/ty-1
Y-141	R	R	N/A	ty-3/ty-3	ty-3/ty-3	ty-2/ty-2	ty-1/ty-1
Y-142	R	R	Ty-3/ty-3	Ty-3/ty-3	Ty-3/ty-3	ty-2/ty-2	ty-1/ty-1
Y-145	R	R	N/A	ty-3/ty-3	ty-3/ty-3	ty-2/ty-2	ty-1/ty-1
Y-152	R	R	Ty-3/ty-3	Ty-3/Ty-3	Ty-3/Ty-3	ty-2/ty-2	ty-1/ty-1
Y-154	R	R	N/A	Ty-3/Ty-3	Ty-3a/Ty-3a	ty-2/ty-2	ty-1/ty-1
Y-172	R	R	Ty-3/ty-3	Ty-3/ty-3	Ty-3/ty-3	Ty-2/Ty-2	ty-1/ty-1
Y-180	R	R	N/A	ty-3/ty-3	ty-3/ty-3	ty-2/ty-2	ty-1/ty-1
Y-181*	R	R	N/A	Ty-3/Ty-3	ty-3/ty-3	ty-2/ty-2	ty-1/ty-1
Y-182	R	R	N/A	Ty-3/Ty-3	Ty-3/Ty-3	ty-2/ty-2	ty-1/ty-1
Y-193	R	R	N/A	ty-3/ty-3	ty-3/ty-3	ty-2/ty-2	ty-1/ty-1
Y-195	R	R	N/A	ty-3/ty-3	ty-3/ty-3	ty-2/ty-2	ty-1/ty-1
Y-196	R	R	N/A	ty-3/ty-3	ty-3/ty-3	ty-2/ty-2	ty-1/ty-1
Y-199	R	R	N/A	ty-3/ty-3	ty-3/ty-3	ty-2/ty-2	ty-1/ty-1
Y-200	R	R	N/A	ty-3/ty-3	ty-3/ty-3	ty-2/ty-2	ty-1/ty-1
Y-201	R	R	N/A	ty-3/ty-3	ty-3/ty-3	ty-2/ty-2	ty-1/ty-1
Y-202	R	R	N/A	ty-3/ty-3	ty-3/ty-3	ty-2/ty-2	ty-1/ty-1
Y-203	R	R	Ty-3/ty-3	Ty-3/Ty-3	Ty-3/Ty-3	ty-2/ty-2	ty-1/ty-1
Y-204	R	R	N/A	ty-3/ty-3	ty-3/ty-3	ty-2/ty-2	ty-1/ty-1

Y-208	R	R	Ty-3/ty-3	Ty-3/Ty-3	Ty-3/Ty-3	ty-2/ty-2	ty-1/ty-1
Y-211	R	R	N/A	ty-3/ty-3	ty-3/ty-3	ty-2/ty-2	ty-1/ty-1
Y-228	R	R	Ty-3/ty-3	Ty-3/Ty-3	Ty-3/Ty-3	ty-2/ty-2	ty-1/ty-1
Y-232	R	R	Ty-3/ty-3	Ty-3/Ty-3	Ty-3/Ty-3	ty-2/ty-2	ty-1/ty-1
Y-243	R	R	N/A	ty-3/ty-3	ty-3/ty-3	ty-2/ty-2	ty-1/ty-1
Y-244	R	R	N/A	ty-3/ty-3	ty-3/ty-3	ty-2/ty-2	ty-1/ty-1
Y-258	R	R	Ty-3/Ty-3	Ty-3/Ty-3	Ty-3/Ty-3	ty-2/ty-2	ty-1/ty-1
Y-264	R	R	N/A	Ty-3/Ty-3	Ty-3a/Ty-3a	ty-2/ty-2	Ty-1/Ty-1
Y-270	R	R	N/A	Ty-3/Ty-3	Ty-3a/Ty-3a	ty-2/ty-2	ty-1/ty-1
Y-279	R	R	Ty-3/ty-3	ty-3/ty-3	Ty-3/Ty-3	ty-2/ty-2	ty-1/ty-1
Y-290	R	R	N/A	Ty-3/Ty-3	Ty-3a/Ty-3a	ty-2/ty-2	ty-1/ty-1
Y-292	R	R	Ty-3/ty-3	Ty-3/ty-3	Ty-3/ty-3	ty-2/ty-2	ty-1/ty-1
Y-301	R	R	N/A	Ty-3/Ty-3	Ty-3a/Ty-3a	ty-2/ty-2	ty-1/ty-1
Y-302	R	R	N/A	Ty-3/Ty-3	Ty-3/Ty-3	ty-2/ty-2	ty-1/ty-1
Y-306	R	R	N/A	ty-3/ty-3	ty-3/ty-3	ty-2/ty-2	ty-1/ty-1
Y-310	R	R	N/A	ty-3/ty-3	ty-3/ty-3	ty-2/ty-2	ty-1/ty-1
Y-317*	R	R	N/A	Ty-3/Ty-3	ty-3/ty-3	ty-2/ty-2	ty-1/ty-1
Y-318	R	R	N/A	ty-3/ty-3	ty-3/ty-3	ty-2/ty-2	ty-1/ty-1
Y-326	R	R	N/A	Ty-3/Ty-3	Ty-3a/Ty-3a	ty-2/ty-2	ty-1/ty-1
Y-329	R	R	N/A	Ty-3/Ty-3	Ty-3/Ty-3	ty-2/ty-2	ty-1/ty-1
Y-331	R	R	N/A	Ty-3/Ty-3	Ty-3/Ty-3	ty-2/ty-2	ty-1/ty-1
Y-334	R	R	N/A	ty-3/ty-3	ty-3/ty-3	ty-2/ty-2	ty-1/ty-1
Y-340	R	R	N/A	Ty-3/Ty-3	Ty-3a/Ty-3a	ty-2/ty-2	ty-1/ty-1
Y-342	R	R	N/A	Ty-3/Ty-3	Ty-3/Ty-3	ty-2/ty-2	ty-1/ty-1
Y-343	R	R	N/A	ty-3/ty-3	ty-3/ty-3	ty-2/ty-2	ty-1/ty-1
Y-347*	R	R	N/A	Ty-3/Ty-3	ty-3/ty-3	ty-2/ty-2	ty-1/ty-1
Y-355	R	R	N/A	ty-3/ty-3	ty-3/ty-3	ty-2/ty-2	ty-1/ty-1
Y-371	R	R	N/A	Ty-3/Ty-3	Ty-3a/Ty-3a	ty-2/ty-2	ty-1/ty-1
Y-377	R	R	N/A	ty-3/ty-3	ty-3/ty-3	ty-2/ty-2	ty-1/ty-1
Y-379	R	R	N/A	ty-3/ty-3	ty-3/ty-3	ty-2/ty-2	ty-1/ty-1
Y-384	R	R	N/A	Ty-3/Ty-3	Ty-3/Ty-3	ty-2/ty-2	ty-1/ty-1
Y-387	R	R	N/A	ty-3/ty-3	ty-3/ty-3	ty-2/ty-2	ty-1/ty-1
Y-390	R	R	N/A	Ty-3/Ty-3	Ty-3a/Ty-3a	ty-2/ty-2	Ty-1/Ty-1
Y-427	R	R	N/A	Ty-3/Ty-3	Ty-3a/Ty-3a	ty-2/ty-2	ty-1/ty-1

R= Resistance
N/A= Molecular screening not performed
P2017.4= April 2017 Phenotypic observation
P2017.9= September 2017 phenotypic observation
M2017.4= Molecular screening performed in April 2017
M2017.9= Molecular screening performed in September 2017.
*recombinants breeding lines as revealed by ACY and P6-25 markers

Description: Field characterization and molecular screening of tomato breeding lines stocked in Green Port Company using the newly developed ACY marker and the previously published markers P6-25, SCAR1 and SCAR2. Although all the breeding lines have displayed resistance on field during two consecutive growing seasons against TYLVD, certain have carried TYLCD resistance genes Ty-1/Ty-3 and Ty-2 and others have not. This might be due to experiment error or those hybrids may harbor another resistance gene such as Ty-4, Ty-5 or Ty-6. (TableS1).

Table S4: Hybrid varieties selected during the period of two consecutive phenotypic observation and molecular analysis assays.

Hybrid varieties	Field evaluation		MS P6-25	MS ACY
	P2017.4	P2017.9	M 2017.4	M 2017.9
12cwi-47	R	R	Ty-3a/ty-3a	Ty-3/ty-3
13cy-133	R	R	Ty-3a/ty-3a	Ty-3/ty-3
12cwi-165	R	R	Ty-3a/ty-3a	Ty-3/ty-3
14c2r-11	R	R	Ty-3a/ty-3a	Ty-3/ty-3
14c2r-23	R	R	Ty-3a/ty-3a	Ty-3/ty-3
14c2r-4	R	R	Ty-3a/ty-3a	Ty-3/ty-3
MT-2199	R	R	Ty-3a/ty-3a	Ty-3/ty-3
14c2r-3	R	R	Ty-3a/ty-3a	Ty-3/ty-3
14c2r-27	R	R	Ty-3a/ty-3a	Ty-3/ty-3
MT-2025	R	R	Ty-3a/ty-3a	Ty-3/ty-3
14r-94	R	R	Ty-3a/ty-3a	Ty-3/ty-3
14r-117	R	R	Ty-3a/ty-3a	Ty-3/ty-3
14r-95	R	R	Ty-3a/ty-3a	Ty-3/ty-3
14r-87	R	R	Ty-3a/ty-3a	Ty-3/ty-3
FS-560	R	R	Ty-3a/ty-3a	Ty-3/ty-3
14r-82	R	R	Ty-3a/ty-3a	Ty-3/ty-3
AG208	R	R	Ty-3a/ty-3a	Ty-3/ty-3
14c2r-8	R	R	Ty-3a/ty-3a	Ty-3/ty-3
15r-19	R	R	Ty-3a/ty-3a	Ty-3/ty-3
15r-108	R	R	Ty-3a/ty-3a	Ty-3/ty-3
15r-138	R	R	Ty-3a/ty-3a	Ty-3/ty-3
15r-43	R	R	Ty-3a/ty-3a	Ty-3/ty-3
15r-122	R	R	Ty-3a/ty-3a	Ty-3/ty-3
15r-67	R	R	Ty-3a/ty-3a	Ty-3/ty-3
15r-106	R	R	Ty-3a/ty-3a	Ty-3/ty-3
15r-53	R	R	Ty-3a/ty-3a	Ty-3/ty-3
15r-167	R	R	Ty-3a/ty-3a	Ty-3/ty-3
15r-123	R	R	Ty-3a/ty-3a	Ty-3/ty-3
15r-35	R	R	Ty-3a/ty-3a	Ty-3/ty-3
15r-32	R	R	Ty-3a/ty-3a	Ty-3/ty-3
15r-25	R	R	Ty-3a/ty-3a	Ty-3/ty-3
15r-86	R	R	Ty-3a/ty-3a	Ty-3/ty-3
15r-16	R	R	Ty-3a/ty-3a	Ty-3/ty-3
15r-39	R	R	Ty-3a/ty-3a	Ty-3/Ty-3
15r-44	R	R	Ty-3a/ty-3a	Ty-3/ty-3
15r-22	R	R	Ty-3a/ty-3a	Ty-3/Ty-3
15r-76	R	R	Ty-3a/ty-3a	Ty-3/ty-3
15r-69	R	R	Ty-3a/ty-3a	Ty-3/ty-3
15p-22	R	R	Ty-3a/ty-3a	Ty-3/ty-3

15p-144	R	R	Ty-3a/ty-3a	Ty-3/ty-3
15p-160	R	R	Ty-3a/ty-3a	Ty-3/ty-3
15p-148	R	R	Ty-3a/ty-3a	Ty-3/ty-3
15p-142	R	R	Ty-3a/ty-3a	Ty-3/ty-3
15p-118	R	R	Ty-3a/ty-3a	Ty-3/ty-3
15p-131	R	R	Ty-3a/ty-3a	Ty-3/ty-3
15p-133	R	R	Ty-3a/ty-3a	Ty-3/ty-3
AG1330	R	R	Ty-3a/ty-3a	Ty-3/ty-3
13t-73	R	R	Ty-3a/ty-3a	Ty-3/ty-3
AG158	R	R	Ty-3a/ty-3a	Ty-3/ty-3
15p-205	R	R	Ty-3a/ty-3a	Ty-3/ty-3
13t-70	R	R	Ty-3a/ty-3a	Ty-3/ty-3
14t-9	R	R	Ty-3a/ty-3a	Ty-3/ty-3
14t-6	R	R	Ty-3a/ty-3a	Ty-3/ty-3
14t-2	R	R	Ty-3a/ty-3a	Ty-3/ty-3
13cy-123	R	R	Ty-3a/ty-3a	Ty-3/ty-3
13cy-107	R	R	Ty-3a/ty-3a	Ty-3/ty-3

P2017.4= Phenotypic observation by April 2017, P2017.9= Phenotypic observation by September 2017, M2017.4= Molecular screening performed in April 2017, M2017.9= Molecular screening performed in September 2017. MS P6-25 = Molecular screening using *Ty-3* linked marker P6-25, MS ACY= Molecular screening using ACY marker

Description: Field characterization and molecular screening of tomato hybrid varieties developed in Green Port Company using the newly developed ACY marker and the previously published marker P6-25. The resistant hybrids were selected during two consecutive field screening assay against TYLCV. Molecular screening test were further performed, which detected allelic resistance genes *Ty-3a* and *Ty-3* as revealed by the markers P6-25 and ACY, respectively. (TableS3)

```
                  ************************************** ***********************
Ty-IS     GATGGCCGCGCCTTTTCTTTTTATGTGGTCCCCACGAGGGTTCCACAGACGTCACTGTCAACCAAT
Ty-Mld    GATGGCCGCGCCTTTTCCTTTTATGTGGTCCCCACGAGGGTTACACAGACGTCACCGTCAACCAAT
Ty-Gs     GATGGCCGCGCCTTTTCCTTTTATGTGGTCCCCACGAGGGTTCCACAGACGTCACTGTCAACCAAT
Ty-MMN    GATGGCCGCGCCTTTTCTTTTTATGTGGTCCCCACGAGGGTTCCACAGACGTCACTGTCAACCAAT
Ty-JT     GATGGCCGCGCCTTTTCTTTTTATGTGGTCCCCACGAGGGTTCCACAGACGTCACTGTCAACCAAT
Ty-GP     GATGGCCGCGCCTTTTCCTTTTATGTGGTCCCCACGAGGGTTCCACAGACGTCACTGTCAACCAAT

          * ************************ *************************** ***** ********
Ty-IS     CAAATTGCATCCTCAAACGTTAGATAAGTGTTCATTTGTCTTTATATACTTGGTCGCCAAGTATTTTGTCTTGCAATATG
Ty-Mld    CAAATTGCATCCTCAAACGTTAGATAAGTTTTCATTTGTCTTTATATACTTGGTCCCCAAGTAGTTTGTCTTGCAATATG
Ty-Gs     CAAATTGCATCCTCAAACGTTAGATAAGTTTTCATTTGTCTTTATATACTTGGTCCCCAAGTTTTTTGTCTTGCAATATG
Ty-MMN    CAAATTGCATCCTCAAACGTTAGATAAGTTTTCATTTGTCTTTATATACTTGGTCCCCAAGTATTTTGTCTTGCAATATG
Ty-JT     CAAATTGCATCCTCAAACGTTAGATAAGTTTTCATTTGTCTTTATATACTTGGTCCCCAAGTAGTTTGTCTTGCAATATG
Ty-GP     CGAATTGCATCCTCAAACGTTAGATAAGTTTTCATTTGTCTTTATATACTTGGTCCCCAAGTAGTTTGTCTTGCAATATG

          ********************************************************************
Ty-IS     TGGGATCCACTTCTAAATGAATTTCCTGAATCTGTTCACGGATTTCGTTGTATGTTAGCTATTAAATATTTGCAGTCCGT
Ty-Mld    TGGGACCCACTTCTAAATGAATTTCCTGAATCTGTTCACGGATTTCGTTGTATGTTAGCTATTAAATATTTGCAGTCCGT
Ty-Gs     TGGGACCCACTTCTAAATGAATTTCCTGAATCTGTTCACGGATTTCGTTGTATGTTAGCTATTAAATATTTGCAGTCCGT
Ty-MMN    TGGGACCCACTTCTTAATGAGTTTCCTGAATCTGTTCACGGATTTCGTTGTATGTTAGCTATTAAATATTTGCAGTCCGT
Ty-JT     TGGGATCCACTTCTAAATGACTTTCCTGAATCTGTTCACGGATTTCGTTGTATGTTAGCTATTAAATATTTGCAGTCCGT
Ty-GP     TGGGATCCACTTCTAAATGACTTTCCTGAATCTGTTCACGGATTTCGTTGTATGTTAGCTATTAAATATTTGCAGTCCGT

          ********************************************************************
Ty-IS     TGAGGAAACTTACGAGCCCAATACATTGGGCCACGATTTAATTAGGGATCTTATATCTGTTGTAAGGGCCCGTGACTATG
Ty-Mld    TGAGGAAACTTACGAGCCCAATACATTGGGCCACGATTTAATTAGGGATCTTATATCTGTTGTAAGGGCCCGTGACTATG
Ty-Gs     CGAGGAAACTTACGAGCCCAATACATTGGGCCACGATTTAATTAGGGATCTTATATCTGTTGTAAGGGCCCGTGACTATG
Ty-MMN    TGAGGAAACTTACGAGCCCAATACATTGGGCCACGATTTAATTAGGGATCTTATATCTGTTGTAAGGGCCCGTGACTATG
Ty-JT     TGAGGAAACTTACGAGCCCAATACATTGGGCCACGATTTAATTAGGGATCTTATATCTGTTGTAAGGGCCCGTGACTATG
Ty-GP     TGAGGGAACTTACGAGCCCAATACATTGGGCCACGATTTAATTAGGGATCTTATATCTGTTGTAAGGGCCCGTGACTATG

          *************************************************************************
Ty-IS     TCGAAGCGACCAGGCGATATAATCATTTCCACGCCCGTCTCGAAGGTTCGCCGAAGGCTGAACTTCGACAGCCCATACAG
Ty-Mld    TCGAAGCGACCAGGCGATATAATCATTTCCACGCCCGTCTCGAAGGTTCGCCGAAGGCTGAACTTCGACAGCCCATACAG
Ty-Gs     TCGAAGCGACCAGGCGATATAATCATTTCCACGCCCGTCTCGAAGGTTCGCCGAAGGCTGAACTTCGACAGCCCATACAG
Ty-MMN    TCGAAGCGACCAGGCGATATAATCATTTCCACGCCCGCCTCGAAGGTTCGCCGAAGGCTGAACTTCGACAGCCCATACAG
Ty-JT     TCGAAGCGACCAGGCGATATAATCATTTCCACGCCCGCCTCGAAGGTTCGCCGAAGGCTGAACTTCGACAGCCCATACAG
Ty-GP     TCGAAGCGACCAGGCGATATAATCATTTCCACGCCCGCCTCGAAGGTTCGCCGAAGGCTGAACTTCGACAGCCCATACAG
```

Fig.S1 Comparison of the 390bp DNA sequences between six different strain of TYLCV; Ty-IS = TYLCV-Israel strain [IR:Boj:28-2], Ty-Mld=TYLCV-Isolate Mld, Ty-Gs=TYLCV-Isolate Goseong Ty-MMN=TYLCV-Isolate Mu_Mu5-N, Ty-JT= TYLCV-IsraelJapanTosa2005, Ty-GP= TYLCV-Green Port.

Description:

The obtained sequence from the cloned 360bp amplicon was aligned with other five different TYLCV isolate. DNA sequence from Israel and Japan isolates showed more than 98% of sequence homology with DNA sequence from TYLCV-green port isolates (the DNA sequence of the infected Ty virus detected in our Company).

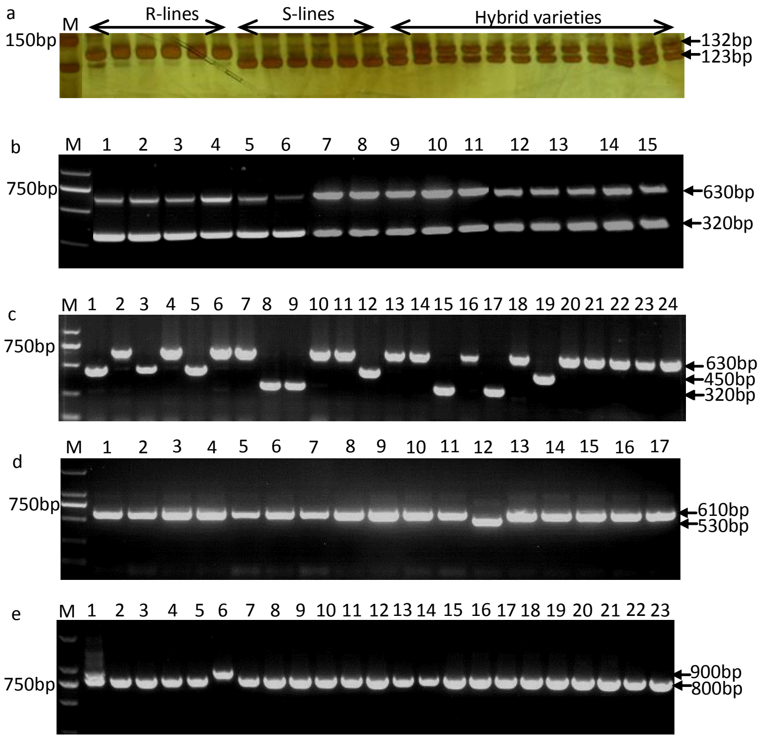

Fig S2. Molecular screening results of the hybrid varieties and breeding lines; (a) Molecular screening of breeding lines (R-lines=resistant lines and S-lines= susceptible lines) and hybrid varieties using ACY, where 132bp indicates the presence of resistance genes either *Ty-1*,*Ty-3* or *Ty-3a* and 123bp indicates the presence of susceptible gene *ty-1*, *ty-3* or *ty-3*; (b) Molecular screening of hybrid varieties using P6-25, where 630bp indicates the presence of resistance gene *Ty-3a* and 320bp the presence of susceptible gene *ty-3*; (c) Molecular screening of breeding lines using P6-25, where 630bp indicates the presence of resistance gene *Ty-3a*, 450bp the presence of resistance gene *Ty-3*, and 320bp the presence of susceptible gene *ty-3*; (d) Molecular screening of breeding lines using SCAR1, where 530bp indicates presence of resistance gene *Ty-1* and 610bp the presence of susceptible gene *ty-1*; (e) Molecular screening of breeding lines using SCAR2, where 900bp indicates the presence of resistance gene *Ty-2* and 800bp the presence of susceptible one *ty-2*; M= marker

Description: Molecular screening of tomato breeding lines and hybrid varieties stocked in Green Port Company using the newly developed ACY marker and the previously published markers P6-25, SCAR1 and SCAR2. Here, we found some of our stocked tomato varieties and breeding lines carrying TYLCD resistance genes Ty-1/Ty-3 and Ty-2 (FigS1).
